# Activation of volume-sensitive Cl^−^ channel mediates autophagy-related cell death in myocardial ischaemia/reperfusion injury

**DOI:** 10.18632/oncotarget.10050

**Published:** 2016-06-14

**Authors:** Yuesheng Xia, Yan Liu, Tong Xia, Xing Li, Cong Huo, Xin Jia, Lin Wang, Rong Xu, Ning Wang, Mingming Zhang, Hong Li, Xiaoming Wang

**Affiliations:** ^1^ Department of Geriatrics, Xijing Hospital, Fourth Military Medical University, Xi'an, China; ^2^ Department of Preventive Medicine, Sun Yat-sen University, Guangzhou, China; ^3^ Department of Cardiology, Xijing Hospital, Fourth Military Medical University, Xi'an, China

**Keywords:** ischemia/reperfusion injury, VSOR Cl^−^ channel, reactive oxygen species, autophagy, cell death, Pathology Section

## Abstract

Excessive reactive oxygen species (ROS) plays an important role in myocardial ischemia/reperfusion (I/R) injury, which triggers not only myocardial cellular apoptosis but also autophagy-related cell death, in which volume-sensitive outwardly rectifying (VSOR) Cl^−^ channel-activated by ROS contributes to cell apoptotic volume decrease, playing an incipient incident of cellular apoptosis. However, whether VSOR Cl^−^ channel concurrently participates in autophagy-related cell death regulation remains unclear. To illuminate the issue, studies underwent in myocardial vitro and vivo I/R model. Rats were performed to ischemia 30 minutes and subsequent reperfusion 24-96 hours, ROS scavenger (NAC), VSOR Cl^−^ channel blocker (DCPIB) and autophagy inhibitor (3MA) were administered respectively. Results showed that oxidative stress, LC3-II stain and inflammation in myocardial tissue were markedly increased, lysosome associated membrane protein-2 (LAMP2) were significantly reduced with I/R group as compared with sham group, reperfusion significantly led to damage in myocardial tissue and heart function, whereas the disorder could be rescued through these agents. Moreover, primary neonatal rat cardiomyocytes hypoxia/reoxygenation model were administered, results showed that VSOR Cl^−^ channel-activated by reoxygenation could cause both cell volume decrease and intracellular acidification, which further increased LC3 and depleted of LAMP2, resulting in autophagy-related cell death. Interestingly, VSOR Cl^−^ channel-blocked by DCPIB could stably maintain the cell volume, intracellular pH, abundant LAMP2 and autophagic intensity regardless of ROS intension derived from reoxygenation injury or adding H_2_O_2_. These results first demonstrate that VSOR Cl^−^ channel-activated is a pivotal event to trigger autophagy-related death, which reveals a novel therapeutic target to decrease myocardial I/R injury.

## INTRODUCTION

Myocardial infarction is the leading cause of death worldwide and continues to rise, early restoration of coronary blood flow plays an important role in minimizing myocardial tissue injury through thrombo­lytic therapy, coronary artery bypass grafting or primary percutaneous interven­tion [1, 2]. However reperfusion may contribute to newer myocardial injury called as myocardial ischemia/reperfusion (I/R) injury, in which oxidative damage plays a critical role to initiate excess reactive oxygen species (ROS) generation and eventually progresses to cell necrotic, apoptotic and autophagic cell death [3–5]. Current anti-apoptosis has been largely reported to protect the heart from I/R injury, however increasing evidence indicates that modulation of autophagy is a novel therapeutic strategy in myocardial I/R injury [6–8].

Autophagy is an evolutionarily conserved intracellular lysosomal degradative process to eliminate long-lived proteins and damaged organelles, which involves segregation of double membrane-bound autophagosomes that fuse with lysosomes and degraded by lysosomal enzymes then recycled as a source of energy in response to diverse conditions stress. A basal autophagic pathway activity is necessary to maintain cellular energy homeostasis and macromolecular synthesis during nutrient deprivation and various forms of cellular stress, however, autophagic dysfunction may contribute to numerous health issues, including neurological degeneration, cardiomyopathies, cancer, diabetes, pathologies of aging and I/R injury [8–11]. Autophagy-induced by various stresses has been gradually determined, any barriers in the autophagic process are likely to lead to dysfunction of mitochondrial autophagy, for instance suppression of constitutive cardiomyocyte autophagy, or impairment of late stages of autophagy in the absence of lysosome associated membrane protein-2 (LAMP2) in patients with Danon disease and in mice with LAMP-2 ablation, resulting in cardiomyopathy. LAMP2 is a critical determinant of autophagosome-lysosome fusion, LAMP2 knockdown causes cardiomyocyte autophagosome accumulation which leads to autophagy-related cell death [11–14]. Previous viewpoint has generally been considered that autophagy has distinct functions in myocardial I/R process, which acts both beneficial cardioprotective during ischemia stress and myocardial injury in subsequent reperfusion, however it becomes very clear now that increased autophagic flux is beneficial regardless during ischemia or reperfusion. Obviously, according to current view, reperfusion injury is derived from LAMP-2 depletion, therefore we should focus on impaired autophagosome clearance, which may trigger excessive ROS, causes fragmentary autophagy. Importantly, the autophagic response is closely connected with the dynamic change of ROS in myocardial ischemia/reperfusion.

ROS is a powerful activator of autophagy in myocardial reperfusion [4–7, 15]. Physiological level ROS is essential signaling molecules, however, excessive ROS generation may affect the autophagic function, leading to disorder of cellular homeostasis and organ functioning [16–18]. Meanwhile autophagy may promote cell death by degrading the antioxidant enzyme catalase, resulting in ROS production increased and depleted of LAMP2, which initiate ROS-induced ROS release and a pernicious cyclic feedback [15, 17, 19–21], leading to autophagosome accumulation, oxidative burden and acute inflammation, affecting cellular homeostasis, thus ultimately accelerate the progress of autophagic cardiomyocyte death [11, 14]. These indicate that impaired autophagosome clearance may increase ROS levels and trigger mitochondrial permeabilization leading to a necrotic mechanism of cell death in cardiac I/R injury, in which the change of cell morphological characteristics is decidedly associated with cell death.

Apoptosis, autophagy-related death and necrosis are the major processes of mammalian cell death. Cell volume changes have been used as one of the key discriminators between apoptosis and necrosis, which are associated with persistent whole-cell shrinkage and swelling, respectively. The regulatory volume decrease (RVD) observed soon after cell swelling is accomplished by parallel activation of volume-sensitive outwardly rectifying (VSOR) Cl^−^ channel in numerous cell types [22–24]. On the other hand, non-swelling-coupled activation of VSOR Cl^−^ channel has been reported to cause apoptosis volume decrease (AVD) in many cells [25–28]. During heart I/R process, ATP-deficient rapidly appear in ischemia phase, the RVD process fail to be activated due to VSOR Cl^−^ channel-inhibited by elevation of the intracellular free Mg^2+^ level [29], however, during reperfusion process, VSOR Cl^−^ channel-activated by excessive ROS generation can induce AVD. Maeno et al showed that AVD induction and RVD facilitation induced by TNF-αor STS were prevented by pretreatment with blockers of VSOR Cl^−^ channels such as 4-(2-butyl-6,7-dichlor-2-cyclopentylindan-1-on-5-yl) oxybutyric acid (DCPIB), a selective inhibitor of VSOR Cl^−^ channel [30]. Current evidence and our previous studies showed that inhibition of VSOR Cl^−^ channel could suppress the apoptotic events [26, 31–34]. Meanwhile these reveal a pivotal role of ROS in the activation of VSOR chlorine channel in response to both swelling and non-welling stimuli cardiomyocytes. Undoubtedly, ROS triggers the AVD process, is an early prerequisite to apoptosis by activating VSOR Cl^−^ channel, however the detailed signaling pathway for VSOR Cl^−^ activation and AVD induction has not been clarified.

VSOR Cl^−^ channel presents ubiquitously expressed in a wide variety of mammalian cells including cardiomyocytes, which is the main anion channel in cytomembrane, its structure equipped with LRRC8A is gradually revealed [35]. VSOR chlorine currents are sensitive to chlorine channel blockers. Our previous study revealed that DCPIB could suppress cardiomyocyte apoptosis through inhibition of VSOR Cl^−^ channel-activated [33, 34]. However, no evidence is currently available regards to the role of VSOR Cl^−^ channel in autophagy-related cell death regulation, and whether autophagy participates in regulation of cell volume has not been elucidated. We hypothesize that VSOR Cl^−^ channel-activated by ROS triggers cell volume change, which is associated with autophagy-related cell death. To confirm this hypothesis, studies undergo in myocardial vitro and vivo I/R model to reveal whether VSOR Cl^−^ channel mediates autophagic cardiomyocyte fate.

## RESULTS

### Inhibited VSOR Cl^−^ channel restores cardiac function and protects rat from myocardial I/R injury

**Figure 1:**
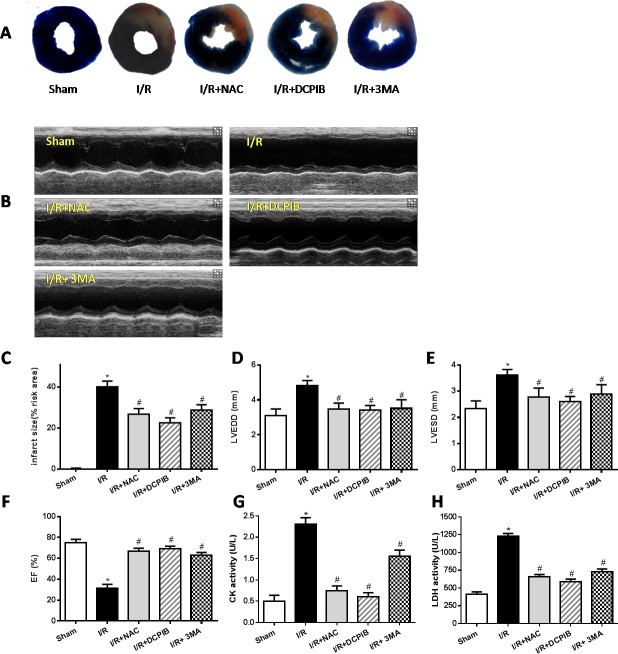
Inhibition of VSOR Cl^−^ channel, ROS and autophagy restore cardiac function and rescue myocardial injury after I/R The LV function and myocardial infarct were determined after 24 hours of reperfusion. **A.** Myocardial infarct size images; **B.** Representative echocardiographic images; **C.**-**H.** The infarct area, myocardial enzyme and echocardiographic analyses. *P < 0.05 compared with sham group; ^#^*P* < 0.05 compared with I/R group.

To explore whether VSOR Cl^−^ channel-activated by ROS production was relevant to autophagic cell death in myocardial I/R injury, the I/R injury model was administered by occluding the left anterior descending coronary artery (LAD) as our described previously [36]. SD rat hearts were subjected to ischemia(30min) followed by reperfusion (24h), ROS scavenger (N-acetyl-l-cysteine, NAC), blocker of VSOR Cl^−^ channel (DCPIB) and inhibitor of autophagy (3-Methyladenine, 3-MA) were injected intraperitoneally 10 min before reperfusion respectively. The results showed that myocardial infarct size in I/R group was 35.6 ± 1.74% significantly higher than the sham group (1.06 ± 0.08%) (*P* < 0.05, *n* = 6), however, treatment with DCPIB, NAC and 3MA significantly reduced the infarct areas compared with I/R group (*P* < 0.01, Figure 1A, 1C). These data indicate that ROS scavenger, inhibitors of VSOR Cl^−^ channel and autophagy could reduce cardiac infarct lesions in I/R rat model.

To determine whether DCPIB, NAC and 3-MA treatment also improved the cardiac functions in myocardial I/R model. Echocardio-graphy was employed to measure main cardiac function as described previously [37]. The results showed that left ventricular end-systolic (LVESD) and left ventricular end-diastolic (LVEDD) in I/R group were 3.53±0.16mm and 4.7±0.12mm, respectively, significantly higher than that in the sham group (*P* < 0.05, *n* = 6 Figure 1B, 1D, 1E), notably, DCPIB, NAC and 3-MA treatment significantly reduced these index. Meanwhile, EF (31.6±3.4%) in I/R group were also reduced compared with the sham group (73.0±3.3%), inhibitors and ROS scavenger treatment significantly increased these index (Figure 1B, 1F) (*P* < 0.05, *n* = 6). These indicate that DCPIB treatment not only reduces infarct lesions but also improves cardiacfunction in I/R-induced heart failure.

The activity of lactate dehydrogenase (LDH) and creatine kinase (CK) levels in serum are considered as myocardial damage marker enzymes. Compared with sham group, the levels of CK, LDH in I/R group were increased (*P* < 0.01). However, inhibitors and ROS scavenger pretreatment significantly reduced the I/R-induced increase in myocardial LDH and CK release in rat heart (*P* < 0.05, Figure 1G, 1H).

### Inhibition of VSOR Cl^−^ channel restrains inflammation and oxidative stress

**Figure 2:**
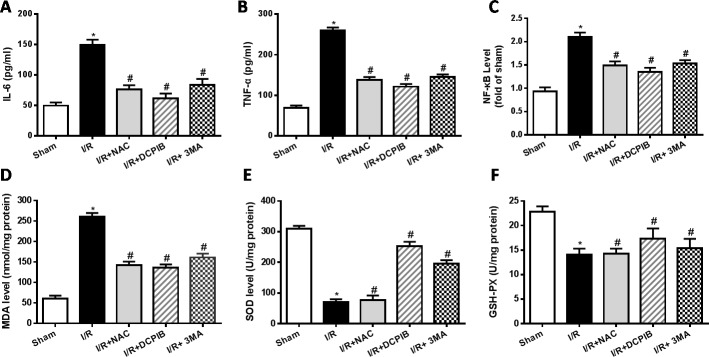
Inhibition of VSOR Cl^−^ channel, ROS and autophagy reduce rat serum inflammation and suppress myocardial oxidative stress after I/R *P < 0.05 compared with sham group; ^#^*P* < 0.05 compared with I/R group.

The inflammatory reaction and oxidative stress present important role in the myocardial ischemia-reperfusion injury [40]. The serum levels of IL-6, tumor necrosis factor-α (TNF-α) and nuclear factor-κB (NF-κB) were detected by the enzyme-linked immunosorbent assay (ELISA) technique according to the instruction of the ELISA Kit. Results showed that pro-inflammatory cytokines including IL-6, TNF-α and NF-kB in serum were significantly increased in the I/R group compared with sham group (*P* < 0.05). In contrast, inhibitors and ROS scavenger pretreatment significantly decreased the levels of pro-inflammatory cytokines (*P* < 0.05. Figure 2A, 2B, 2C). These data suggest that ROS scavenger, inhibitors of VSOR Cl^−^ channel and autophagy may decrease the release of pro-inflammatory cytokines in myocardial I/R injury.

Mitochondria are the major site of ROS production in mammalian cells, H_2_O_2,_ a relatively stable diffusible molecule, is the main composition of ROS, which can cross membranes and can be produced from other ROS, ultimately it is removed by GSH peroxidase. Oxidative stress is believed to be elevated in heart I/R, the content of malondialdehyde (MDA) is an index of peroxidation, it can indirectly reveal the level of ROS generation. The superoxide dismutase (SOD), MDA and glutathione/glutathione disulide (GSH-PX) concentrations were measured according to the instruction of the kits. The present data showed that activities of GSH-PX and SOD were decreased significantly in the I/R group as compared with the sham group (*P* < 0.05). However, DCPIB and 3MA treatment reduced signifi­cant elevation of GSH-PX and SOD activities compared with the I/R group (*P* < 0.05, Figure 2E, 2F). The content of MDA increased significantly after myocardial I/R injury (*P* < 0.05, Figure 2D), however ROS scavenger and inhibitors treatment sig­nificantly decreased the MDA content com­pared with the I/R group (*P* < 0.05, Figure 2D). These data suggest that autophagy also play an important role in inflammatory and oxidative stress, especially DCPIB elevates the activity of antioxidant enzymes and decreases the ROS generation.

### Blocking VSOR Cl^−^ channel contributes to a continuous abundance of LAMP-2 through inhibition of ROS, intracellular acidification and autophagy

**Figure 3:**
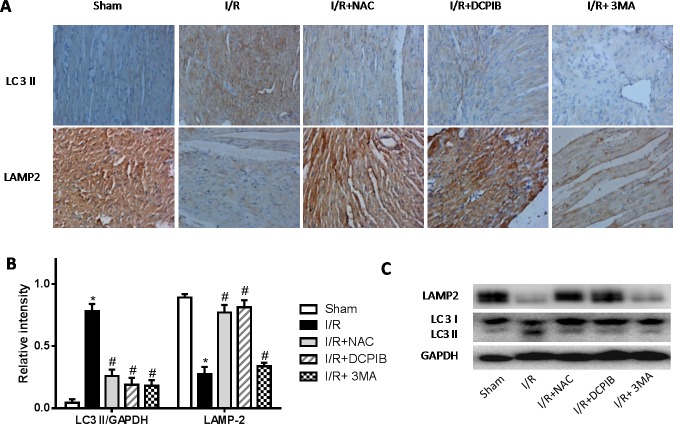
Inhibition of VSOR Cl^−^ channel, ROS and autophagy with NAC, DCPIB and 3MA respectively markedly restrain LC3 level, and stanchly maintain LAMP-2 abundance **A.** Immunohistochemical images (×400); **B.**, **C.** Western blot analysis. *P < 0.05 compared with sham group; ^#^*P* < 0.05 compared with I/R group. These data demonstrate those inhibitors, especially both DCPIB and NAC distinctly inhibit rapid decline in LAMP2 abundance and immoderate soaring LC3 derived from reperfusion injury.

**Figure 4:**
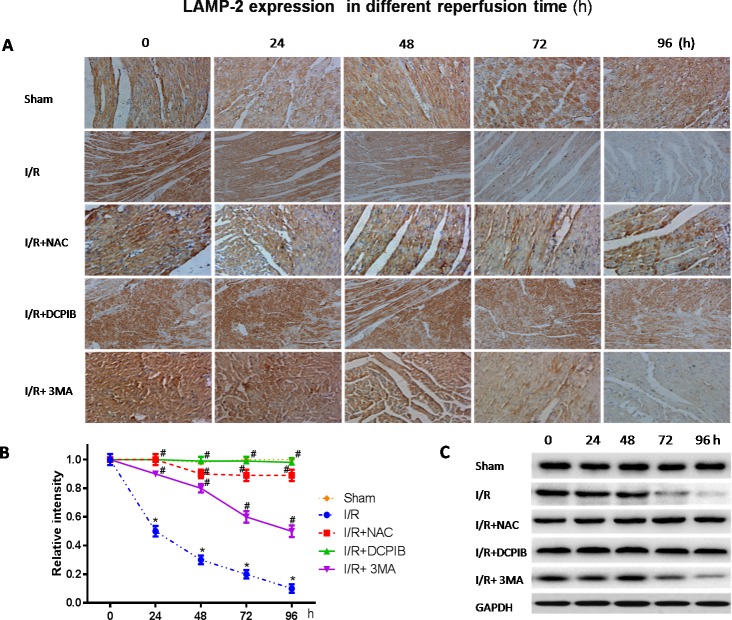
Blockate of VSOR Cl^−^ channel and ROS stably interdict deplete of LAMP2 abundance contribute from I/R at each time **A.** Immunohistochemical images (×400); **B.**, **C.** Western blot analysis. *P < 0.05 compared with sham group; ^#^*P* < 0.05 compared with I/R group. These data demonstrate a decline in LAMP2 abundance distinctly derived from reperfusion injury following to time, and being reversed by DCPIB and NAC.

**Figure 5:**
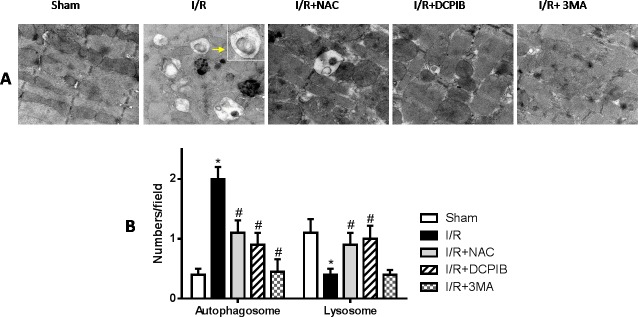
Blockate of VSOR Cl^−^ channel, ROS and autophagy with NAC, DCPIB and 3MA respectively markedly restrain generated autophagosome, and the first two stanchly maintain lysosome abundance **A.** Transmission electron microscopy (TEM) images(×12600); **B.** TEM analysis. *P < 0.05 compared with sham group; ^#^*P* < 0.05 compared with I/R group.

Persistent evidence shows that myocardial autophagy is increasing in ischemic process, and further increased in following reperfusion, which triggers mitochondrial permeabilization leading to a necrotic mechanism in cardiac reperfusion injury. The depressed incident is largely due to the generation of ROS, which contributes to autophagosome abundance and insufficient clearance, in return further increased ROS levels, which is involved in a noteworthy decline in LAMP2 in myocardial reperfusion [11]. To explore the relationship between LAMP-2, microtubule-associated protein1 light chain 3 (LC3) expression and those inhibitors in myocardial I/R physiopathology (Figure 3), immunohistochemical analysis was administered as described previously [37], Anti-LAMP2a and anti-LC3 II antibodies were used for the detection of lysosomes and autophagic intensity, respectively. Rat subjected to I/R revealed a manifest decline in LAMP2 and increasing autophagic intensity (*P* < 0.01; Figure 3), meantime greater numbers of autophagosomes were observed in cardiomyocytes from I/R mice than from inhibitors-treated mice, and those changes were significant with the prolongation of reperfusion time (*P* < 0.01; Figure 3 and 4). These data were the first evidence that blocked of VSOR Cl^−^ channel may attenuate adverse cardiac I/R damage through unremitting maintainability of LAMP-2 expression regardless of the prolongation of reperfusion time (*P* < 0.01, Figure 4). Rat subjected to I/R revealed a remarkable decline in LAMP2 abundance and increasing LC3II, however, comparative analysis revealed that both NAC, DCPIB and 3MA were significantly stabilize the expression of LAMP2 and decrease LC3 in heart tissues (*P* < 0.01; Figure 3 and 4). These results indicate that the VSOR Cl^−^ channel has a previously unrecognized role in autophagic death regulation. 3MA is well-characterized by inhibition of autophagy, rarely affects ROS products, presents a validity of autophagic inhibition and relatively weak stability in LAMP2 (Figure 3 and 4). Interestingly, numbers of LAMP-2 express in I/R group presented significantly different during the time frame of reperfusion, whereas NAC and DCPIB stably maintained the numbers of LAMP-2 dots (*P* < 0.01; Figure 3). Moreover, electron microscopic observation revealed the autophagosomes were rarely seen in cardiomyocytes from rat received inhibitors and ROS scavenger compared to the rat I/R group, these typical autophagosomes contain intracellular organelles, such as degraded mitochondria and membrane-like structures (Figure 5). These studies indicate that VSOR Cl^−^ channel-activated in I/R is associated with conspicuous depleting of LAMP2, which contributes to myocardial I/R injury through further enhance ROS generation, and relevant inhibitors and ROS scavenger may reverse the event.

### VSOR Cl^−^ channel-activated by ROS triggers autophagy-related cell death in myocytes hypoxia / reoxygenation injury

**Figure 6:**
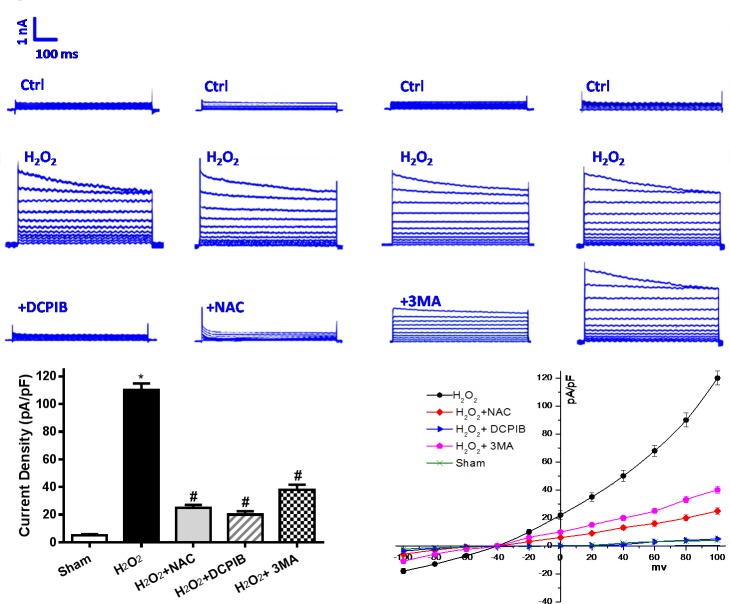
Activation of VSOR Cl^−^ channel by I/R is associated with excessive reactive oxygen species (ROS) and autophagy Reoxygenation-induced ROS is mainly composed of H_2_O_2_. Increased VSOR Cl^−^ currents in H_2_O_2_ exposed cardiomyocytes present a classic simulate I/R model. H_2_O_2_-induced VSOR Cl^−^ currents were inhibited by adding DCPIB (10 μM); NAC (15 mM) and 3MA (15 μM), *n* = 5 for each group. Negligible background Cl^−^ currents recorded under isosmotic solution (Ctrl). H_2_O_2_-induced Cl^−^ currents exhibiting representative properties of VSOR Cl^−^ currents. Background Cl^−^ currents and H_2_O_2_-induced VSOR Cl^−^ currents were similer in each group. So we primarily appraise the relationship for the mean current densities of each group after intervention. These data reveal activation of VSOR Cl^−^ channel by H_2_O_2_, is related to ROS, and being reversed by DCPIB, NAC and 3MA. *P < 0.05 compared with sham group; ^#^*P* < 0.05 compared with I/R group.

To verify whether the autophagy-related cell death was mediated by VSOR Cl^−^ channel-activated. We first determined that VSOR Cl^−^ channel-activated by ROS could contribute to autophagosome accumulation in myocardial ischemia/reperfusion injury, which has been reminded *in vivo* study. One potential target seems to focus on the outwardly rectifying volume-stretch-sensitive Cl^−^ current (I_Cl,Vol_). Previous and our studies have demonstrated that activation of ROS production by osmotic swelling, as well as exogenous H_2_O_2_, can elicit I_Cl,Vol_ in ventricular myocytes [33, 34]. Hereby, H_2_O_2_ (10μM) was employed to simulate I/R injury for I_Cl,Vol_ formation, meanwhile we administrated DCPIB (50μM), NAC (15mM) and 3MA (15μM) to primary neonatal rat cardiomyocytes. Whole-cell recordings revealed that H_2_O_2_ (10μM) could activate I_Cl,Vol_ with the similar peak amplitude, these currents were outwardly rectifying with a time-dependent inactivation at positive potentials, however, ROS scavenger and VSOR Cl^−^ channel inhibitor could significantly inhibit I_Cl,Vol_ by 80.1% ± 4.7% and 94.5% ± 5.1%, respectively, on the other hand, 3MA, an autophagic inhibitor by inhibition of class III phosphatidylinositol 3-kinase, inhibited I_Cl,Vol_ by 37.31 ± 2.30%. VSOR Cl^−^ current also exhibited relative higher level when exposed 3MA (*P* < 0.01; Figure 6). Nevertheless ROS scavenger and all of these inhibitors increased myocardial cell survival (*P* < 0.01; Figure 8C).

**Figure 7:**
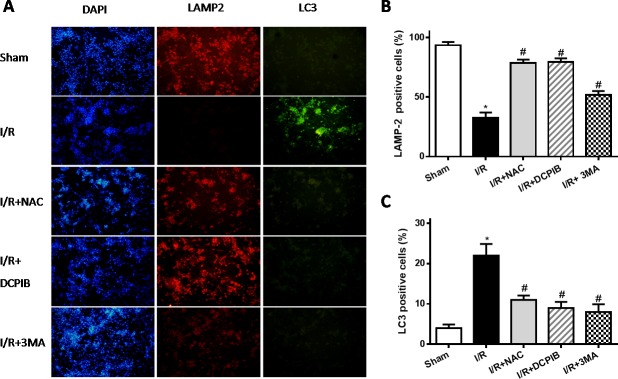
Blockate of VSOR Cl^−^ channel and ROS stably maintain LAMP2 abundance and restrain excess autophagy contribute from reoxygenation **A.** Representative images of immunofluorescent staining; *n* = 5 for each group. (×100); **B.**,**C.** staining cell analysis. *P < 0.05 compared with sham group; ^#^*P* < 0.05 compared with I/R group.

**Figure 8:**
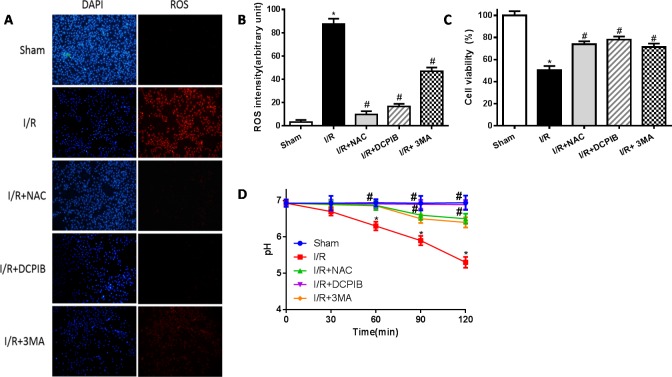
Blockate of VSOR Cl^−^ channel may restrain excess ROS and stabilize intracellular pH contribute from reoxygenation **A.** Immunofluorescent staining images; *n* = 5 for each group (×400); **B.** staining cell analysis; **C.** Cell viability analysis by the MTT-assay; **D.** intracellular pH analysis. *P < 0.05 compared with sham group; ^#^*P* < 0.05 compared with I/R group.

Primary neonatal rat cardiomyocytes with hypoxia for 6 hours followed by reoxygenation for 2 hours, DCPIB (50μM), NAC (15mM) and 3MA (15μM) were administrated to these cells before reoxygenation, then assessed the contribution of VSOR Cl^−^ channel to the LAMP2 in reoxygenation cardiomyocytes by immunofluorescence. Meanwhile intracellular pH were measured as described previously [38]. Results showed that reoxygenation significantly increased ROS and LC3, reduced LAMP2 expression and intracellular pH, myocardial cell survival reduced markedly to compare with sham group; however in ROS scavenger and inhibitor agents groups, ROS and LC3 reduced markedly, LAMP2 increased 4-9-fold, restoration of intracellular pH and myocardial cell survival were increased compare with hypoxia/reoxygenation (simulate I/R) group, (*P* < 0.01; Figures 7 and 8). Overall our data showed that reoxygenation changed intracellular pH, resulted in intracellular acidification, which further activated autophagy. These suggested VSOR Cl^−^ channel-activated by ROS resulted in accumulation of autophagosome and intracellular acidification due to depleting of LAMP2, the pernicious disorder contributed to autophagy-related cell death.

### Blocking of VSOR Cl^−^ channel contributes to partial Beclin-1 inhibition in myocardial hypoxia-reoxygenation injury

**Figure 9:**
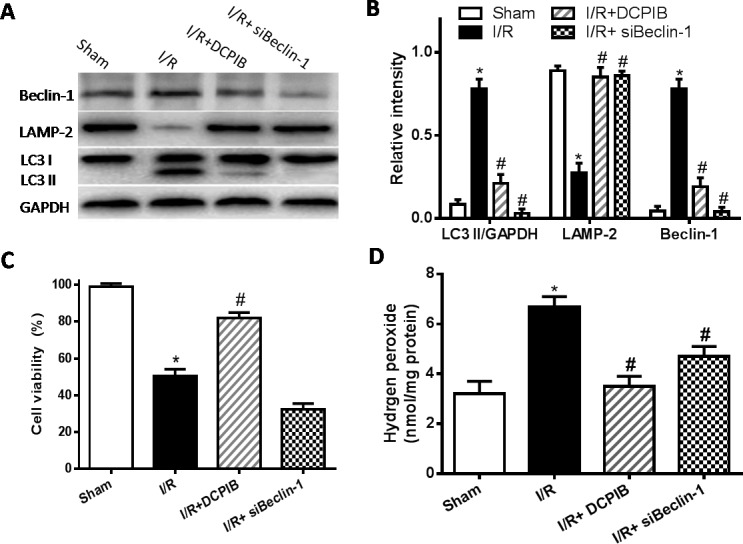
VSOR Cl^−^ channel blockers causes partial Beclin-1 inhibition in myocardial hypoxia-reoxygenation injury **A.**, **C.** Western blot analysis; **B.** Cell viability was measured by the MTT-assay; **D.** intracellular H_2_O_2_ analysis. *P < 0.05 compared with sham group; ^#^*P* < 0.05 compared with I/R group.

**Figure 10:**
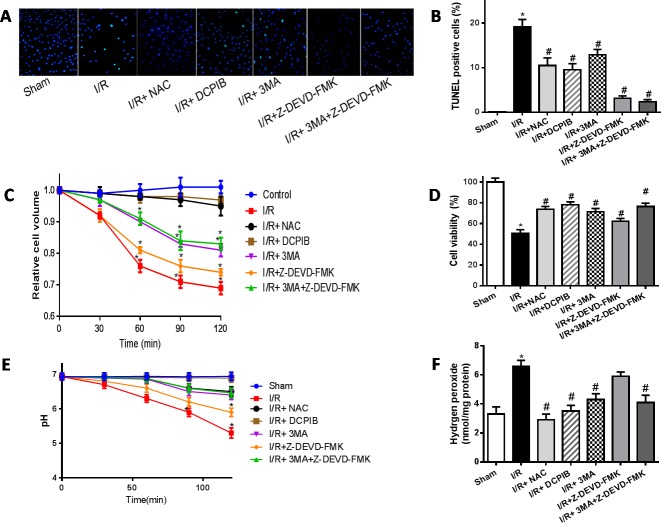
The time courses of changes in the mean cell volume **A.**,**B.** TUNEL staining images and analysis (×400); **C.**,**D.** Cell volume and cardiomyocyte viability analysis; **E.** intracellular pH analysis; **F.** intracellular H_2_O_2_ analysis. *P < 0.05 compared with sham group; ^#^*P* < 0.05 compared with I/R group.

Together with our above studies, our results suggest that blocking of VSOR Cl^−^ channel may protect heart from I/R damage by its intricate pathways. Reperfusion injury impairs autophagosome clearance, contributing to increased cardiomyocyte death. Beclin-1 is a pivotal protein in autophagic activity [39], whether blocking VSOR Cl^−^ channel may prevent myocardial impairment through regulation of Beclin1 is fully unknown. To test the hypothesis, knockdown Beclin-1 by adenovirus transducing Beclin-1-siRNA (for 48 hours) and subjected to hypoxia-reoxygenation. Intracellular H_2_O_2_ was monitored as previously described method [40]. The results showed that Beclin-1-siRNA completely inhibited the autophagosome formation, whereas reduced myocardial survival (*P* < 0.05, Figure 9). Contrary to those, blocking VSOR Cl^−^ channel with DCPIB, partially inhibited Beclin-1 contributed to significant cardioprotection (*P* < 0.05, Figure 9). These data reveal that increasing levels of Beclin-1 by reoxygenation may aggravate the impairment in autophagosome clearance, while it is well known essential autophagy play a key role in cell survival, knockdown of Beclin-1 destroy the autophagic base line, contributing to increase cardiomyocyte death. Moreover, previous study revealed cardiomyocytes were susceptible to apoptosis induced by inhibition of autophagy, beclin1 knockdown significantly increased caspase-3 expression in I/R [20]. However, our data showed that blocking of VSOR Cl^−^ channel could inhibit cells apoptosis and excessive autophagy (Figure 10A). These results suggest blocking of VSOR Cl^−^ channel may contribute a double benefit, which prevent myocardial impairment through inhibition of apoptosis and autophagy-related cell death.

### VSOR Cl^−^ channel-activated triggers autophagic volume decrease contributing to autophagy-related cell death

**Figure 11:**
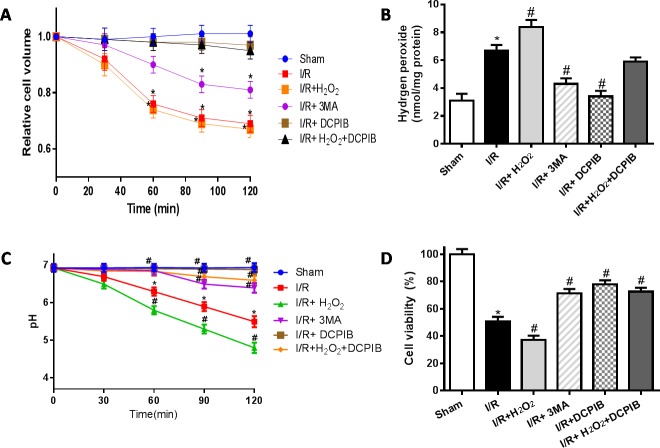
VSOR Cl^−^ channel-activated directly triggers cell volume decrease, when lockate of VSOR Cl^−^ channel, even adding ROS, which failed to cause cell volume decrease in reoxygenation **A.**,**D.** Cell volume and cardiomyocyte viability analysis; **C.** intracellular pH analysis; **B.** intracellular H_2_O_2_ analysis. *P < 0.05 compared with sham group; ^#^*P* < 0.05 compared with I/R group.

ROS has been previously implicated in autophagy and autophagy-related cell death, however, up to now, the typical morphological characteristics of autophagic cell death remains unclear. Cell death such as apoptotic and necrotic cell death are associated with alterations of cell volume regulation, the control of cell volume is essential not only for maintaining physiological cell activities but also for cell survival. The RVD may be caused by activation of VSOR Cl^−^ channel in many mammalian cell [22, 24, 30]. Moreover, the RVD is abolished when a blocker of VSOR Cl^−^ channel DCPIB is added [30]. We attempted to determine whether autophagy-related cell death induced by VSOR Cl^−^ channel-activated was associated with cell volume change. Cell volume was measured as described previously [41]. Our present results showed that blocking the volume-regulatory Cl^−^ channel or autophagy could prevent the RVD induction, whereas inhibitor of caspase-3, Z-DEVD-FMK (100 μM), failed to prevent the AVD induction (P < 0.01; Figure 10), as AVD is an upstream event of these biochemical apoptotic events. Nevertheless inhibited apoptosis and autophagy could further increase myocardial cell survival (Figure 10D). Intriguingly, blocking of VSOR Cl^−^ channel could stanchly maintain the cell volume regardless of ROS intension derived from reoxygenation injury or H_2_O_2_ added, ROS alone seems uncertainty to possess the critical determinants of induced AVD in the presence of DCPIB(*P* < 0.01; Figure 11). These results suggested that autophagy-related cell death could be rescued by blocking VSOR Cl^−^ channel due to preventing the early-phase autophagic cell shrinkage and intracellular acidification. The present study first reveal that ROS mediates autophagy-related cell death through activation of VSOR Cl^−^ channel.

## DISCUSSION

Myocardial tissue reperfusion after ischemia is essential for cardiac function recovery in heart attack and stroke, however it causes reperfusion injury due to the generation of mitochondrial ROS [2, 4, 15, 18, 19]. In cardiomyocytes, oxygen is mainly reduced by two pathways: one is the mitochondrial electron transport system, which reduces more than 90% of oxygen to H_2_O, another is processed by intracellular enzymes to produce ROS [19]. It has generally been considered that I/R cause metabolic disturbance, oxidative damage and aberrant immune responses through ROS production. Under normal conditions, endogenous ROS play important roles in modulating normal cellular processes, however subsequent reperfusion drives extensive ROS generation by reverse electron transport at mitochondrial complex I [42]. Importantly, numerous studies showed that cardiomyocyte damage induced by ROS has been proved to be largely due to the disorder of autophagy [18–20]. Our present study also showed that ROS scavenger may protect heart from I/R injury through autophagic regulation.

Autophagy is a physiological process in which a cell digests its own constituents through the lysosomal degradative pathway. Under both basal and stressful conditions, autophagy promotes cell survival through maintaining needed levels of ATP production and protein synthesis, which derive from degraded membrane lipids and proteins within autophagosomes [6–8]. In the heart, autophagy occurs constitutively in the normal myocardium but is substantially increased in several heart diseases including heart failure, hypertrophy, ischemic cardiomyopathy and myocardial ischemia-reperfusion injury. Inefficient autophagy or its absence leads to poor myocardial performance, and inhibition of starvation-induced autophagy results in cardiac dysfunction and dilatation [9, 10]. During myocardial I/R injury, the myocardium is subjected to sudden ischemia just as extreme starvation, which induces autophagy may contribute to the survival of cardiomyocytes, whereas reperfusion provokes superabundant autophagosome formation, simultaneously markedly declines in LAMP2 abundance, resulting in autophagosome accumulation in cardiomyocytes [11]. Consistent with accumulating studies demonstrate ROS drives the progress from autophagic protection to autophagic death in myocardial ischemia/reperfusion, which seems to reach a consensus. Notably, disruption of autophagic pathway are likely to lead to dysfunction of autophagy, for instance suppression of constitutive cardiomyocyte autophagy, or impairment of late stages of autophagy in the absence of LAMP2 in patients with Danon disease [11, 13, 14]. Our study also provides strong evidence that myocardial I/R injury results in excessive ROS generation, which obviously depletes of LAMP2 abundance, contributing to autophagosome accumulation, consequently leading to autophagy-related cell death. Unexpectedly, the impairment may be reversed through blocking of VSOR Cl^−^ channel and ROS.

Definite evidence determines that activation of VSOR Cl^−^ channel is concerned with cardiac contractile dysfunction [22, 24, 25], however DCPIB may attenuate cardiomyocyte apoptosis and rescue cardiac function by inhibition of VSOR Cl^−^ channel-activated [26–29]. The present study proposes a novel evidence that VSOR Cl^−^ channel-activated by ROS may cause autophagic cell shrinkage, leading to intracellular acidification and autophagy-related cell death. In addition, single ROS fails to achieve the effect in absent of VSOR Cl^−^ channel-activated. It reveals that VSOR Cl^−^ channel-activated by ROS seems to open the Pandora Box, which triggers not only apoptosis, but also autophagy-related cell death.

Autophagy and apoptosis are two major stress-response pathways that are often co-regulated but elicit opposite cellular outcomes, they share in the common upstream signalling components, inhibition of either pathway can also result in the activation of the other. For instance blocking apoptosis in cells can trigger autophagy, whereas blocking autophagy would rapidly induce apoptosis [20]. Surprisingly, the present study showed that blocking-VSOR Cl^−^ channel could concurrently restrain apoptosis and autophagy-related cell death, the prominent response should focus on VSOR Cl^−^ channel, VSOR Cl^−^ channel-activated causes large amounts of chloride ions outflows lead to intracellular acidification, the acidic environment violently increase autophagic levels, which aggravates the depleting of LAMP2. Therefore, it is possible that both autophagy and apoptosis modulate cell death independently, trend to which death pathway-activated depends largely on the status of cell and the intracellular signaling environment. Another Beclin1 is a well-conserved protein and plays a central role during the autophagy process, which is initially identified as the anti-apoptotic protein, binds Bcl-2 during normal conditions to inhibit autophagosome formation. However, upon stress, beclin1 is released from Bcl-2 and can then proceed to perform its function in autophagy [20, 43]. Our data indicate that blocking of VSOR Cl^−^ channel present a maintainability of LAMP-2 abundance and fractional decline in Beclin-1, which contributes a double benefit to protect myocardial myocardium from apoptosis and autophagic cell death.

However, even administer experimental animal and cell models in present study, it could not establish clearly whether another or more signal channels participate in cardiac I/R injury [44–46]. Herein, we at least demonstrate that Cl^−^ Channel-activated by ROS triggers cardiac autophagy-related cell death. In the present study, for the first time, we demonstrate a novel ROS terminal responsive mechanism, whereby activation of Cl^−^ channel triggers both apoptosis and autophagy-related cell death. Furthermore, under blocking of VSOR chloride channel, even increased ROS by adding H_2_O_2_, nevertheless ROS alone failed to increase autophagy strength in cardiac I/R stress.

## CONCLUSIONS

Our results first demonstrate that activation of VSOR Cl^−^ channel blocks autophagosome clearance due to LAPM2 reduction, leading to autophagy-related cell death and myocardial I/R injury, which is related to cell volume decrease and intracellular acidification. These findings reveal a novel ROS terminal responsive mechanism in controlling of cell death, meanwhile demonstrating that inhibition of VSOR Cl^−^ channel-activated is a potential therapeutic target to decrease myocardial ischaemia/reperfusion injury.

## MATERIALS AND METHODS

### Reagents and antibodies

DCPIB, NAC, 5-bromo-2-deoxyuridine (BrdU), MTT, 3-MA, antimouse and anti-rabbit secondary antibodies were purchased from Sigma-Aldrich (St. Louis, USA); LC3B, Beclin-1, GAPDH and LAMP2 primary antibodies were purchased from Genetex (Abcam, England); Antibodies against Beclin1 was obtained from Sigma-Aldrich (St. Louis, USA). Dihydroethidium (DHE) was from Molecular Probes (Eugene, OR, USA). LC3II primary antibody and Z-DEVD-FMK were purchased from Santa Cruz Biotechnology (Santa Cruz, CA, USA); Terminal Deoxynucleotidyltransferase- mediated dUTP Nick End Labeling (TUNEL) was from Roche Applied Science (SandhoferStrasse, Mannheim, Deutschland).

### Animals

Male Sprague-Dawley rats (250-350g; 8 weeks old) were purchased from the Laboratory Animal Center of the Fourth Military Medical University (Xi'an, China). Animals received humane care in compliance with the Guidelines for Management of Experimental Animals, which was in accordance with Guide for the Care and Use of Laboratory Animals published by the National Institutes of Health (NIH Pub. 1996). The project was reviewed and approved by the appropriate institutional review committees. Animals were housed with a chow diet and water available adlibitum under diurnal lighting conditions and in a temperature controlled environment until the start of the experiment.

### Myocardial ischemia/reperfusion model and interventions *in vivo*

SD Rats were anesthetized with sodium pentobarbital (Sigma, St. Louis, USA, 60 mg/kg) with mechanically ventilated. Ischemia was performed by reversible LAD coronary ligation with a 6-0 silk suture for 30 minutes, followed by removal of the ligature and reperfusion for 24-96 hours. Rats were randomly assigned to five experimental groups. There were 6 rats in each group: (1) Sham: rats was administered the ligature placed around the LAD but without occlusion, (2) I/R: rats were subjected to I/R, (3) I/R + NAC: rats received I/R plus NAC 130mg/kg, (3) I/R + DCPIB: rats received I/R plus DCPIB 10mg/kg, (4) I/R + 3MA: rats received I/R plus 3MA 0.3mg/kg. Rats underwent 30 min of myocardial ischemia followed by 24-96h of reperfusion. NAC, a ROS scavenger; DCPIB, a selective inhibitor of VSOR chlorine channel and 3MA, a autophagic inhibitor were administered as intraperitoneal injection 10 min before the onset of reperfusion, respectively.

### Myocardial infarct size determination

Infarct size was established by 2, 3, 5-triphenyltetrazolium chloride (TTC, Sigma-Aldrich) staining. The LAD was disengaged after 24 hours of reperfusion, Evans blue dye was injected into the aorta and coronary arteries, the area at risk was determined by staining with 2.0 ml 1% Evans blue. Each heart was excised and cut into 1-mm slices, then slices were incubated in 1 % TTC for 15 min at 37°C. The infarct area (white) and the area at risk (red and white) were determined using an image analyzer (Image-Pro Plus 3.0, Media Cyber-netics, Silver Spring, MD, USA). Infarct size was expressed as a percentage of the risk area volume (%, infarct size/risk area).

### Echocardiography to assess cardiac function

Rats were anesthetized with sodium pentobarbital and cardiac function was measured using echocardiography (VisualSonicsVeVo 770) 24 h after reperfusionas. Cardiac geometry and function were evaluated using 2-D guided M-mode echocardiography equipped with a 15-16 MHz linear transducer. The parameters tested were as follows: LVEF (left ventricular eject fraction), LV end-diastolic (LVEDD) and end-systolic (LVESD).

### Enzyme activity assays

CK and LDH activity was measured to assess the extent of myocardial cell injury. Briefly, samples were collected from the coronary effluent at the end of the experiment, and the activities of LDH and CK were assayed using CK and LDH kits (Nanjing Jiancheng Bio Inst, Nanjing, China).

### Inflammatory cytokines measurement

To investigate the DCPIB in I/R-induced inflammation response, the serum levels of IL-6, TNF-α and NF-κB were detected by the enzyme-linked immunosorbent assay (ELISA) technique according to the instruction of the ELISA Kit (Sigma-Aldrich, St. Louis, MO, USA).

### Oxidative stress assay

After 120 min of the perfusion, hearts were harvested and frozened at −70°C. The hearts were grinded by a liquid nitrogen-chilled tissue pulverizer, weighed the cardiac tissues, and then homogenized in bufer and centrifuged using homogenizer. The SOD, MDA and GSH-PX concentrations were measured according to the instruction of the kits (Nanjing Jiancheng Bio Inst, Nanjing, China).

### Immunohistochemical analysis of LAMP-2 and LC3 expression

Immunohistochemical analysis was administered as described previously [37]. First, 3-mm slices from pretreated myocardium tissue were placed in a bathing solution of 3% H_2_O_2_ and 60% methanol PBS for 30 min and then treated with 0.01 mol/L sodium citrate buffer at 95°C in a microwave oven for 13 min. Then, specimens were treated with 5% normal goat serum and 5% bovine serum albumin in PBS. Before each step, sections were rinsed three times in PBS buffer. Incubation with primary Anti-LAMP-2 (Abcam 1:50) and Anti-LC3 II (Santa Cruz,1:100) antibodies was performed in a PBS-based solution of 1% bovine serum albumin for 12 h at 4°C in the recommended dilutions. After rinsing with PBS, sections were incubated with the corresponding secondary antibodies for 30 min at room temperature. A streptavidin/horseradish peroxidase complex was then applied as a detection system (1:100 dilution) for 1 h. Finally, staining was developed with 3, 39-diaminobenzidine tetra-hydrochloride in 0.05 mol/L Tris-HCl buffer. The primary antibody incubation step was omitted in the negative control. All dates in this study were analyzed by software “Image Pro Plus” (Media Cybernetics Corporation, DC).

### Tissue fixation and transmission electron microscopy

After 24 h reperfusion, left ventricular tissue was fixed for 24 h in 2% paraformaldehyde/2.5% glutaraldehyde, then dissected out at sections 3 mm^3^. Tissue samples were postfixed in 1% osmium tetroxide, stained with 1% uranyl acetate in 0.05 M sodium hydrogen maleate buffer, dehydrated through an ethanol series, and embedded in Agar 100 resin. Ultra-thin sections were stained with 1% uranyl acetate and lead citrate and examined using a Tecnai G2 20 transmission electron microscope.

### Western blot analysis

Protein samples (100 μg) extracted from hearts (*n* = 6 from each group) were subjected to 10% or 15% PAGE and then transferred to PVDF membranes. The membranes were then probed using primary antibodies against LAMP2 and LC3 etc after which the blots were visualized using enhanced chemiluminescence (Amersham). GAPDH served as the loading control.

### Cardiomyocyte hypoxia/ reoxygenation model *in vitro*

Cardiomyocytes were prepared from 1-day-old SD rats and subjected to hypoxia/ reoxygenation. Primary neonatal rat cardiomyocytes were isolated from rat hearts, and ventricles were digested with collagenase II. Subsequently, cardiomyocytes were resuspended in DMEM. Cells were allowed to recover in DMEM culture media for 24 h, followed by a change to serum-free (SF) media to hypoxia (95% N_2_ and 5% CO_2_) for 6 h followed by reoxygenation (95% O_2_ and 5% CO_2_) for 2 h. Before reoxygenation, cardiomyocyte treatments with ROS scavenger: NAC (15 mM) and inhibitor agonists: DCPIB (10 μM), or 3MA (15 μM).

### Whole-cell path-clamp recording

Primary culture of neonatal rat cardiomyocytes were seeded onto 12-mm glass coverslips which were deal with Silicone grease 3-4 times and placed on the bottom of an 0.8-ml bath at a density of 10^6^ cells/ml and incubated 12 h, and then hypoxia (37°C, 5% CO_2_ 95% O_2_ incubator) 1 h prior to electrophysiological studies. Patch-clamp experiments: the VSOR Cl^−^ currents were recorded with an Axon Multiclamp 700B amplifier and Digidata1322A (Axon Instruments, Foster, CA, USA) using the whole-cell conFigureuration. Voltage clamp protocols and data acquisition were controlled by pClamp10 software. Pipettes were fabricated from borosilicate glass capillaries using a micropipette puller (P-2000, Sutter Instrument, Novato, CA, USA) with resistance of 3-5 MΩ when filled with pipette solution. Liquid junction potentials were calculated with JPCalc in pClamp 10 and corrected on-line. For whole-cell recordings, the capacitative transients and access resistance were maximally compensated. The pipette solution (103 mMCsOH, 103 mM Aspartic acid, 25 mMCsCl, 5 mM Mg-ATP, 0.3 mM Na3-GTP, 5 mM EGTA, 10 mM HEPES, and 30 mMmannitol, pH7.4 adjusted with CsOH, 295 mosmol/Kg H_2_O) was used to selectively record whole-cell Cl^−^ currents. The isotonic bathing solution contained 85 mM N-methyl-D-glucamine (NMDG), 85 mMHCl, 10 mM NaCl, 2 mM 4-aminopyridine (4-AP), 2.5 mM BaCl_2_, 0.33 mM NaH_2_PO_4_, 4mM MgCl2, 5 mMTetraethylammonium-Cl (TEA-Cl), 10 mM HEPES, 5.5 mM glucose and 85 mMmannitol (pH7.4 adjusted with NMDG-OH, 305 mosmol/Kg H_2_O). Tetrodotoxin (TTX, 8 μM) and nifidipine (5 μM) were routinely included in bath solutions to block Na^+^ channel and L-type Ca^2+^ channel, respectively. The osmolality of all solutions was measured using a freezing-point depression osmometer (OM802, Vogel, Giessen, Germany).

### Measurements of Intracellular pH

Intracellular pH was measured as the described previously [30] with some modifications. PHluorin as the plasmid, it is a pH-sensitive GFP expressed from plasmid pYES-*P*_ACT1-_pHluorin under control of ACT1 promoter. First, cardiomyocytes were transformed with this plasmid, and transformants were selected in SD medium lacking uracil (SD-Ura) and confirmed by fluorescence microscopy. Cardiomyocytes hypoxia/ reoxygenation in the specified medium were harvested, washed, and resuspended at 1 g of wet cell mass/ml in glucose-free medium Fluorescence intensities at excitation wavelength of 390 and 470 nm were measured at a constant emission wavelength of 512 nm by SpectraMax M3 microplate reader (Molecular Devices).

### Cell volume measurements

Cell volume was measured at room temperature (23-26°C). An electronic cell sizing technique with a Coulter-type cell size analyzer (CDA-500; Sysmex, Kobe, Japan) was employed. The mean volume of the cell population was calculated from the cell volume distribution measured. Suspensions of spherical cells were prepared at different time points of reoxygenation. The standard isotonic solution was principally made of 95 mM NaCl, 1 mM MgCl_2_, 1 mM CaCl_2_, 4.5 mM KCl, 105 mM mannitol and 5 mM HEPES (310 mOsmol/kg-H_2_O).

### Immunofluorescence

After hypoxia/reoxygenation, cells were washed with PBS and fixed with 0.1% paraformaldehyde, then permeabilized with 0.1% Triton X100 and 5% goat serum for 20 minutes at 4°C, and incubated with relevant primary antibodies for 12 h, followed by incubation with a fluorescent secondary antibody (Alexa Fluor). Cells were analyzed for fluorescence using Cell Quest V3.3 software on fluorescence microscope.

### Measurements of intracellular H_2_O_2_

The hydrogen peroxide content in the myocardial mitochondria was measured by colorimetric method as previously described [40], using a commercial kit, based on the reaction with molybdic acid (Jiancheng Biotech Inc., Nanjing, China). Adduct was measured spectrophotometrically at 405 nm in a plate reader (TECAN infinite M200, USA) in strict accordance with manufacturer's instructions.

### TUNEL staining

Apoptosis was assayed by TUNEL staining using the *in situ* TUNEL cell death detection kit according to the manufacturer's instructions. In brief, cells were fixed with 4% paraformaldehyde and permeabilized with 0.3% Triton X-100 for 1h at room temperature, and then washed twice with PBS. Cells were then incubated with the TUNEL assay reaction mixture at 37°C for 1 h, followed by nuclear counterstaining with DAPI. To determine the percentage of apoptotic cells, micrographs of TUNEL-positive nuclei and methyl green-stained nuclei were captured using an Olympus microscope and counted using ImageJ software (Image J version 1.43; NIH) from 10 to 20 random fields at 400 × magnification.

### MTT cell viability assay

Cell viability was assessed by MTT assay. In brief, cardiomyocytes were plated into 96-well culture plates at a density of 5 × 10^4^ /well (100 μl). MTT was added into each well with a final concentration of 0.5 mg/ml, and cells were incubated for 4 h at 37°C. The formazan crystals were dissolved in dimethyl sulfoxide (DMSO, 150 μl/well). The absorbance was detected at 490 nm using a microplate reader (Bio-Rad, Philadelphia, PA, USA).

### Intracellular fluorescence measurement of ROS

Intracellular superoxide was monitored by changes in fluorescence intensity resulting from intracellular probe oxidation. Cardiomyocytes were loaded with 5 μM (DHE for 30 min at 37°C and washed twice with PBS buffer. Cells were captured using a confocal microscope (Nikon).

### RNA interference experiments

RNA interference experiments were performed using small interfering RNAs (siRNAs) and a negative control siRNA (BlockIT: FITC-labeled short RNA with random sequence; Invitrogen, Carlsbad, CA. Briefly, transfection of each siRNA was performed using HiPerFect Transfection Reagent (Quiagen, Hilden, Germany) according to the manufacture's protocol. Transfected cells were rested for 2 days before subjecting to further analysis. Over 80% of cells were found to exhibit FITC fluorescent. Knockdown efficiencies of RNA interference were examined by quantitative RT-PCR.

### Statistical analysis

Values were expressed as mean ± SE. Statistical analysis for a single comparison was performed using Student's *t*-test. Multiple comparisons were performed using one-way ANOVA, followed by the Tukey post-hoc test using SPSS software (IBM SPSS Statistics; version 19, Armonk, NY, USA). *P* < 0.05 was considered as indicating statistical significance.
